# Estrogen Receptor Regulates Male Satellite Cells in a Female, but Not Male, Environment

**DOI:** 10.3390/cells14201606

**Published:** 2025-10-16

**Authors:** Ahmed S. Shams, Brian P. Sullivan, Erik A. Toso, Dawn A. Lowe, Michael Kyba

**Affiliations:** 1 Lillehei Heart Institute and Department of Pediatrics, Medical School, University of Minnesota, Minneapolis, MN 55455, USA; ashams@umn.edu (A.S.S.); tosox012@umn.edu (E.A.T.); 2 Department of Human Anatomy and Embryology, Faculty of Medicine, Suez Canal University, Ismailia 41522, Egypt; 3 Division of Physical Therapy and Rehabilitation Science, Department of Family Medicine and Community Health, Medical School, University of Minnesota, Minneapolis, MN 55455, USA; brian.sullivan2@pennmedicine.upenn.edu (B.P.S.); lowex017@umn.edu (D.A.L.)

**Keywords:** 17β-estradiol, estrogen, estrogen receptor, satellite cells, transplantation, skeletal muscle, regeneration, sex hormones

## Abstract

Skeletal muscle homeostasis is dependent on the satellite cell pool, which is regulated by numerous signaling pathways. Estradiol (E2) function via estrogen receptor alpha (ERα, *Esr1*) plays an important role in satellite cell regulation in females, being necessary for satellite cell maintenance, proliferation and differentiation. Here we investigate this signaling axis in male satellite cells. Male satellite cells express *Esr1* mRNA at similar levels to female satellite cells, and E2 enhances the proliferation of male satellite cell-derived myoblasts in vitro. Deletion of *Esr1* specifically in male satellite cells has no effect on satellite cell number, nor on their ability to self-renew after injury, during regeneration, or when transplanted into male hosts. However, *Esr1* deletion severely reduces self-renewal of male satellite cells when transplanted into female hosts. These data suggest that male satellite cells are competent for E2-ERα signaling, but that this signaling is not efficacious in the male environment, though E2-ERα signaling does become necessary when the male cells are transplanted into a female environment.

## 1. Introduction

Skeletal muscle maintenance and regeneration is dependent on satellite cells [1,2], which express the transcription factor Pax7 [3] and reside between the basal lamina and the sarcolemma of the skeletal muscle myofiber [4]. In the unperturbed condition, satellite cells are deeply quiescent, but upon stimulation (muscle stress or damage), the satellite cells activate and enter the cell cycle, yielding daughter cells that contribute nuclei to muscle fibers or repopulate the satellite cell pool [5,6].

The satellite cell pool is regulated by a complex set of factors in their microenvironment [7,8], in addition to several intrinsic and systemic extrinsic factors that determine the balance between self-renewal vs. myogenic commitment, depth of quiescence, and overall pool size [9,10,11,12,13,14]. Disruption of this harmonic balance and/or signaling leads to loss of the skeletal muscle mass and improper tissue repair in case of damage [15,16,17,18].

Among the best studied systemic signals that change with age are the sex hormones [19,20]. 17β-Estradiol (E2), the predominant and most potent form of estrogen, has a well-described role in skeletal muscle maintenance in females [21]. Consequent to estrogen deficiency in women with ovarian failure, skeletal muscle strength is deleteriously affected [22,23], which has been reversed by hormonal treatment [24,25]. In addition, a significant reduction in the appendicular skeletal muscle mass has been reported in young postmenopausal women [26] with several studies having reported improvement in skeletal muscle mass and composition with hormonal replacement therapy, e.g., in a post-menopausal monozygotic twin controlled study [27,28] and in postmenopausal females [25,29,30]. Furthermore, estrogen-based steroids have long been used as an anabolic agent in the livestock industry [31].

We have previously shown that satellite cells of female mice express *Esr1*, the primary estrogen receptor, and that the homeostatic satellite cell pool size drops 30–70% when mice are subjected to ovariectomy (OVX) or when *Esr1* is conditionally deleted in Pax7-expressing cells of female mice [32,33,34]. Looking at homeostatic satellite cell pool size in humans revealed a positive correlation between PAX7+ cell frequency and E2 levels in perimenopausal women [32]. In mice, the reduction in the satellite cell pool occurs within two weeks of removal of ovarian hormones by OVX, is correlated with apoptosis within the pool [33], and is reversed with E2 treatment [32,34]. Furthermore, we documented a deficient regenerative response to muscle injury in female mice with OVX [32] or *Esr1* KO satellite cells [34]. Thus, both the major female sex hormone, E2, and the primary receptor, ERα, are needed in satellite cells for homeostatic maintenance of the satellite cell pool, and for optimal muscle regeneration in females.

A significant reduction in satellite cell frequency with aging is seen in some muscles [35,36,37,38,39], most strongly the tibialis anterior (TA) and other locomotory muscles, which are also under-exercised in standard laboratory conditions, in both males and females. Less of or no decline is seen in diaphragm and masseter, muscles that are more normally used [40,41]. In the case of females, supplementation with E2 increases the number of satellite cells in the TA and soleus and reverses a subset of age-related gene expression changes [40]. The direct regulation of the satellite cell compartment by E2 in females prompts the question of whether E2 might also regulate satellite cells in males. Males have quite low levels of circulating E2; however, in both males and females, estrogen is produced through the action of aromatase on testosterone, a hormone males have in systemic abundance. Recently overexpression of aromatase in skeletal muscle of male mice led to increased serum and tissue E2 resulting in anabolic effects on muscle [42]. Therefore, it is possible that niche cells expressing aromatase might convert testosterone into locally high levels of E2 in the vicinity of satellite cells in male mice, maintaining the satellite cell pool via E2 independently of systemic levels. In that case, the deletion of *Esr1* in male satellite cells would be predicted to result in a decline in satellite cell number. We previously observed a significant deficit in engraftment when we transplanted WT female satellite cells into OVX hosts compared to sham-treated hosts, as well as when we transplanted *Esr1* KO female cells into WT female recipients compared to *Esr1*-WT female cells into WT female recipients [32]. If male cells were dependent on E2, whether local or systemic, then *Esr1* KO would reduce their engraftment into male as well as female recipients. On the other hand, if satellite cells in muscle of males were intrinsically independent of E2 regulations, the cells should graft equally well into female recipients with or without *Esr1* deletion.

To date the majority of satellite cell transplantation studies have been performed in a sex-matched manner, consequently intrinsic differences or similarities in the response to sex hormones as environmental factors has not been robustly elucidated. Some data exists on fiber differentiation in transplantation assays, and it suggests that WT male and female satellite cells have similar potentials with regard to in vivo differentiation [43], but neither the self-renewal of the stem cell compartment nor its E2 dependence is known.

In the current study we test the hypothesis that estradiol signaling through ERα regulates the male satellite cell pool in a manner comparable to females. We employ in vitro culture of male satellite cell-derived myoblasts in the presence or absence of 17β-estradiol, and we perform sex-matched and reciprocal-sex satellite cell transplantation. Finally, we evaluate the self-renewal potential of male satellite cells with satellite cell-specific temporally regulated genetic ablation of the *Esr1* in the context of transplantation.

## 2. Materials and Methods

### 2.1. Mice

All procedures were performed in accordance with a protocol (2307-41280A) approved (3 November 2023) by the Institutional Animal Care and Use Committees at the University of Minnesota. All experiments were conducted on adult male and female mice 3–6 months of age. Male and female Pax7-ZsGreen mice were used for sex comparison experiments [44]; Esr1^FL/FL^; Pax7CreERT2; Pax7-ZsGreen (experimental), and Esr1^FL/FL^; Pax7-ZsGreen (Control, lacking Cre) mice were generated in-house [32]. Transplant recipients were NSG-mdx^4Cv^ mice [45].

To induce *Esr1* knockout, all mice were treated for five consecutive days with 80 mg/kg IP tamoxifen (Sigma Aldrich, St. Louis, MO, USA) dissolved at a concentration 12.75 mg in 1 mL sunflower oil, after which mice were then maintained on tamoxifen diet (TD.140251 Envigo, Indianapolis, IA, USA) for the remaining duration of each experiment. For the transplantation experiments, recipient mice were treated starting on the day of transplantation. This knockout protocol overcomes the ability of any escapers to reconstitute the satellite cell pool [1,18].

### 2.2. Isolation of Primary Murine Myoblasts

Primary myoblasts were isolated from adult (3–4-month-old) male and female C57Bl6/J mice via lineage depletion using the Miltenyi satellite cell isolation kit, mouse, according to manufacturer’s instructions (Miltenyi Biotec, Gaithersburg, MD, USA, Cat #130-104-268). Briefly, hind limb muscles were dissected, minced in parallel with muscle fibers, and digested in Dispase II and Collagenase type 2 for 30 min at 37 °C. The resulting slurry was manually dissociated, 4 mL of DMEM added and cells filtered through a 100 µM cell strainer. Cells were pelleted via centrifugation at 500× *g* for 5 min. Cells were resuspended in 80 µL of sorting buffer (PBS, pH 7.2, 0.5% BSA, 2 mM EDTA) and 20 µL of the Lin–cell separation microbeads and incubated for 15 min at 4 °C before magnetic separation. Unlabeled cells were collected, centrifuged at 500× *g* for 5 min, resuspended in Myogenic Growth Medium (DMEM/F12 medium (11320033; Thermo Scientific, Waltham, MA, USA) with 20% FBS (PS-FB3, PEAK Serum, Wellington, CO, USA), 10 ng/mL human basic fibroblast growth factor [bFGF; 100-18C; Peprotech, Cranbury, NJ, USA], 1% Pen/Strep [15140122; Gibco], and 1% Glutamax [35050061; Gibco]), and pre-plated for 90 min on an uncoated 10 cm plate at 37 °C and 5% CO_2_. Non-adherent (satellite) cells were transferred to a 0.1% gelatin-coated plate and allowed to proliferate until ≥80% confluent and thereafter considered primary myoblasts.

### 2.3. MTT Proliferation Assay

Mouse primary myoblasts were plated into 0.1% gelatin-coated 96-well plate (4000 cells/well) with phenol red-free F12/DMEM (SH30272.01; Hyclone, Logan, UT, USA) supplemented with 20% CS-FBS (NB036790; Thermo Fisher Scientific), 10 ng/mL human basic fibroblast growth factor, 1% Pen/Strep, and 1% Glutamax. Myoblasts were treated with 17β-Estradiol (E2) or vehicle every 24 h at a concentration of 100 pM (27.24 pg/mL) E2. The MTT assay was performed according to manufacturer’s instructions (11465007001; Roche, Indianapolis, IN, USA). After 48 or 72 h, the MTT labeling reagent [3-(4,5-dimethylthiazol-2-yl)-2,5-diphenyltetrazoliumbromide in PBS] was added to each well (final concentration 0.5 mg/mL) and incubated for 4 h. Cells were solubilized with DMSO and the formazan product measured in a microplate reader at a 570 nm wavelength.

### 2.4. EdU Proliferation Assay

Mouse primary myoblasts were plated into 0.1% gelatin-coated 96-well plate (4000 cells/well) with phenol red free DMEM/F12 (SH30284.01; Hyclone) supplemented with 20% CS-FBS (NB036790; Thermo Fisher Scientific), 10 ng/mL human basic fibroblast growth factor, 1% Pen/Strep, and 1% Glutamax. Myoblasts were treated with E2 or vehicle every 24 h at a concentration of 100 pM (27.24 pg/mL) E2. The Click-iT EdU proliferation assay for microplates was performed according to manufacturer’s instructions (C10499; Thermo Fisher Scientific). After 72 h of treatment, 10 µM EdU was added to cells for 8 h, cells were subsequently fixed, washed, and incubated with the Click-iT reaction cocktail. Cells were then blocked and incubated with the Amplex UltraRed reaction mixture. Fluorescence was subsequently read on a microplate reader with excitation at 568 nm and emission at 585 nm.

### 2.5. Western Blots

Cells were lysed with RIPA buffer supplemented with protease inhibitor cocktail (Complete, Roche), and proteins were separated on 10% SDS-PAGE gels, then transferred to PVDF membranes. Antibodies were diluted in 3% skim milk in TBST and incubated overnight at 4 °C or 1 h at RT. An appropriate HRP conjugated secondary antibody was incubated for 1 h at RT. Membranes were then washed with TBST, and signal was visualized using Pierce ECL Western blotting substrate (Thermo Scientific) for the ER alpha and SuperSignal West Femto Maximum Sensitivity Substrate (Thermo Scientific) for the GAPDH. Antibodies used in the study: GAPDH-HRP (1:5000, 60004, Proteintech), Mouse ER alpha /NR3A1 Antibody (1: 1000, MAB57151 R&D systems)

### 2.6. Flow Cytometry/FACS Sorting

Flow cytometric isolation of satellite cells from bulk hind limb or individual muscle digests (e.g., TA, EDL, Sol, and GC) were performed as described in detail previously [32,41]. Briefly, muscles were dissected, minced in parallel with muscle fibers, and digested with collagenase type II and dispase (17101-015 and 17105-041, resp.; Gibco, Grand Island, NY, USA). For isolation of satellite cells from C57BL/6 mice, mononuclear cells were stained using an antibody mixture of 1 µL PE-Cy7 rat anti-mouse CD31 (clone 390; 561410; BD Biosciences, San Diego, CA, USA), 1 µL PE-Cy7 rat anti-mouse CD45 (clone 30-F11; 552848; BD Biosciences), 1 µL Biotin rat anti-mouse CD106 (clone 429 (MVCAM.A); 553331; BD Biosciences), 1 µL PE Streptavidin (554061; BD Biosciences), and 2 µL alpha7 integrin 647 (clone R2F2; AbLab; Vancouver, BC, Canada). Samples were incubated with antibody cocktail, washed, and resuspended with FACS staining medium (2% Fetal Bovine Serum [FBS; 16000044; Gibco] in phosphate-buffered saline [PBS]) containing 0.5 µg/mL propidium iodide (PI) for analysis on a FACSAriaII SORP (BD Biosciences, San Diego, CA, USA). Total satellite cells (Lin–, i.e., not PE-Cy7+; double-positive, i.e., VCAM1+, Integrin α7+ cells) were analyzed from the entire muscle sample.

To obtain satellite cells from Pax7-ZsGreen mice, mononuclear cells were isolated as described above and were incubated in FACS staining medium containing PI [45]. Absolute satellite cell counts by FACS were confirmed through gating ZsGreen+ cells according to the manufacturer’s instructions. Researchers were blinded during all FACS analyses.

### 2.7. TA Injury and Transplantation

Adult (3–6 months old) Esr1fl/fl; Pax7-ZsGreen male mice carrying Pax7-creERT2 or Cre-negative controls were anesthetized with ketamine and xylazine, both hind limbs shaved and sterilized using surgical betadine solution, the skin over the TA was opened with a scissor, and both TA muscles were exposed. For injury-only studies, 30 µL of 1.2% BaCl2 was injected with a Hamilton syringe and the skin was closed using nonabsorbable suture. For transplantation experiments, 48 h prior to transplantation of cells, 4-month-old recipient NSG-mdx4Cv and C57BL/6J mice were anesthetized with ketamine and xylazine and both hind limbs were subjected to 1200 cGy irradiation using an RS 2000 Biological Research Irradiator (Rad Source Technologies, Inc., Suwanee, GA, USA) with lead shields protecting the body and forelimbs. Twenty-four hours prior to transplant, 15 µL cardiotoxin (10 µM in PBS, Sigma, St. Louis, MO, USA) was injected into the TA under anesthesia as above. Twenty-four hours later, 300 ZsGreen+ satellite cells were collected by FACS from donor mice and transplanted in a volume of 10 µL PBS into each TA. Four weeks after transplantation, TA was prepared for FACS analysis as described above to count the number of ZsGreen+ cells.

### 2.8. RNA Isolation, Reverse Transcription, and qRT-PCR

Total RNA was extracted using a Qiazol reagent (Qiagen) as described in the manufacturer’s protocol. First strand cDNA was generated using the iScript cDNA synthesis kit (BioRad, Hercules, CA, USA). Relative quantification of estrogen receptors was determined by Taqman probes, ERa (ESR1—Mm00433149_m1), ERb (ESR2—Mm00599819_m1), GPER (Mm02620446_s1), and Androgen receptor (Mm0042688_m1) using FAM-based chemistry on a CFX Connect real-time PCR detection system (Bio-Rad). Gene expression was determined with the ∆Ct relative quantification method and shown as relative to *B2M* (Mm00437762_m1), *Tbp* (Mm00446973_m1), *Hprt* (Mm03024075_m1), or *Gapdh* (Mm99999915_g1).

### 2.9. Statistical Analysis

All data were analyzed with two-tailed unpaired Student *t*-tests for determining significant differences between two groups or one-way ANOVA with Tukey’s post hoc for determining significant differences between three or more groups; data was tested for normality using Kolmogorov–Smirnov test. Significance was considered at *p* values of ≤0.05. Data are presented as mean ± SEM. All statistical testing was performed using GraphPad Prism 8.0 (GraphPad Software Inc., San Diego, CA, USA).

## 3. Results

### 3.1. Male Primary Myoblasts Express Esr1 and Are Equally as Responsive to E2 as Female Cells

**Figure 1:**
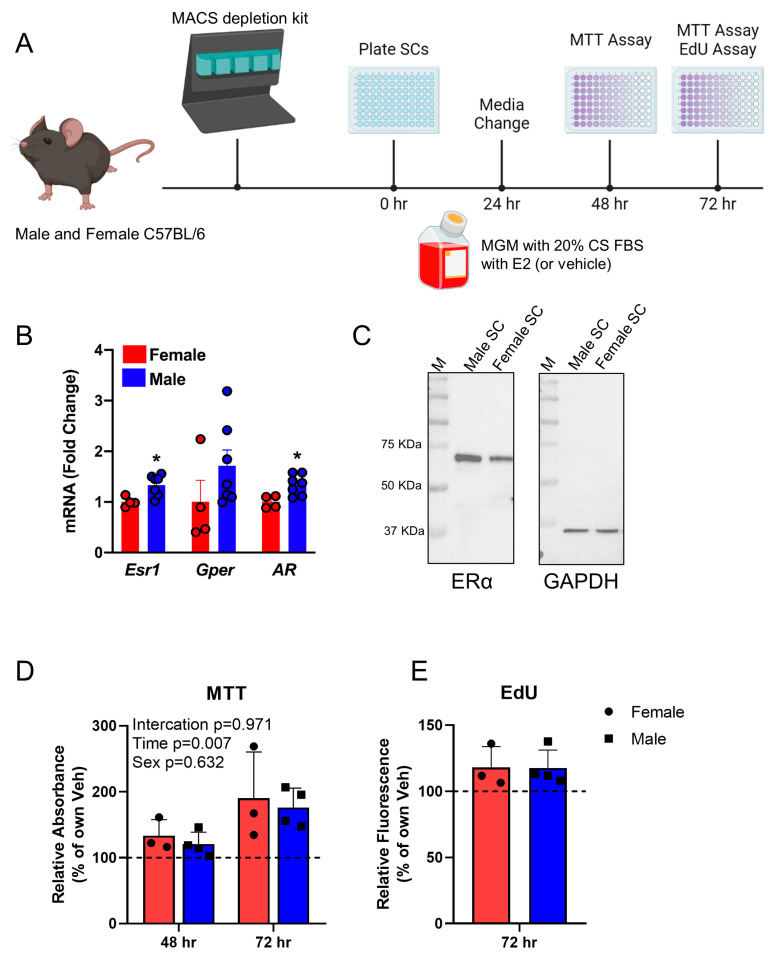
Male and female SCs respond similarly to E2 in vitro. (**A**) Schematic for the in vitro experiment where isolated male and female satellite cells were cultured ± E2 for 48 and 72 h, and analyzed by MTT assay and quantifying EdU+ cells. (**B**) RTqPCR for *Esr1*, *Gper*, and *AR* in satellite cells presented as fold change of the level of expression in the female cells. (**C**) Western blots for ERα and GAPDH control in Lin–Intga7/VCAM1 freshly sorted satellite cells from C57BL/6 male and female mice. (**D**) MTT cell viability assay showing percentage of absorbance relative to the vehicle-treated cells. (**E**) Percentage of EdU incorporation in cells after 72 h in culture with E2, relative to vehicle-treated cells. Charcoal-stripped serum is used to eliminate any background steroid hormones. Data are presented as mean ± SEM, * *p* < 0.05, by *t*-test for (**B**,**E**) and two-way ANOVA for (**D**).

E2 signaling promotes the proliferation of primary myoblasts derived from satellite cells of female mice [34]. To characterize the response of male primary myoblasts to E2, we isolated satellite cells from male and female C57Bl/6J by MACS (Figure 1A). We assessed the expression of the estrogen receptors, ERα and G-Protein coupled estrogen receptor (GPER) (Figure 1B), and found them both expressed at to at least their respective levels in female myoblasts, suggesting potential activity of the pathway. To assess the ERα protein level, we sorted Intga7+/VCAM1+ cells by FACS from male and female C57Bl/6J mice and analyzed by Western blot. We found both male and female satellite cells have detectable ERα protein, slightly higher in male cells, similar to the *Esr1* mRNA results (Figure 1C). Next, we passaged primary myoblasts from male and female (*n* = 4 male mice and 3 female mice) C57BL/6J mice into growth medium with charcoal-stripped serum, to eliminate any influence of background steroid hormones, adding either E2 or vehicle for up to 72 h. We found that male and female satellite cells responded equally well to E2 with regard to proliferation, as measured by MTT assay at 48 and 72 h post plating (Figure 1D). We confirmed this using an independent measure of proliferation, EdU incorporation, and found similar increases in EdU incorporation in male and female cells after 72 h of E2 treatment (Figure 1E).

### 3.2. Male and Female SC Engraft Similarly Regardless of the Sex of the Recipient

**Figure 2:**
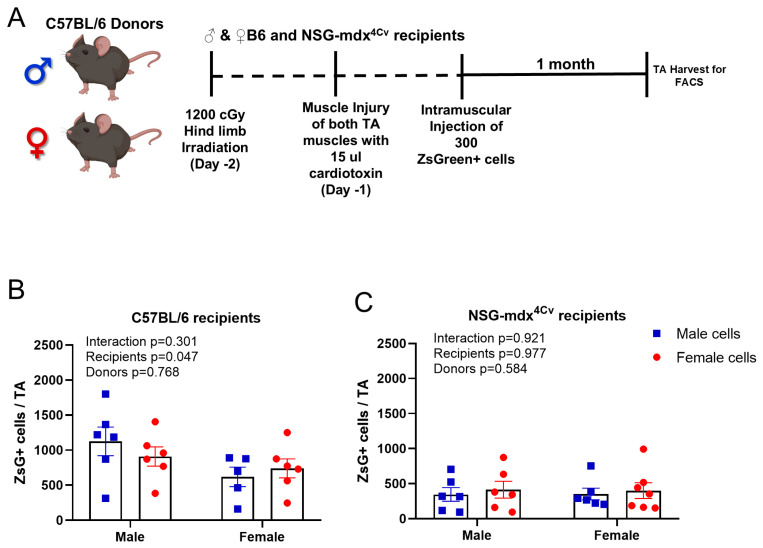
Male and female SCs engraft similarly, regardless of the sex of the recipient. (**A**) Schematic for the transplantation experiment showing the timing of irradiation and TA injury in the recipient mice. (**B**) Total number of Pax7-ZsGreen+ cells in TA muscles of recipient C57 BL/6 mice. Males (*n* = 6). Female (*n* = 6). (**C**) Total number of Pax7-ZsGreen+ cells in TA muscles of recipient NSG-mdx4cv mice. Males (*n* = 6). Female (*n* = 6). Data are presented as mean ± SEM; *p* values were calculated by two-way ANOVA.

We have developed a transplantation-based assay to monitor self-renewal of satellite cells in which 300 Pax7-ZsGreen cells are transplanted into injured, irradiated tibialis anterior (TA) muscles of recipient mice [44,46]. One month after transplantation, the TA muscle is dissected, digested, then analyzed by FACS to count the number of Pax7-Zsgreen cells that self-renewed after transplantation to contribute to the satellite cell pool (Figure 2A and Figure S1A). We employed this assay to investigate the effect of sex as a determinant of engraftment into the satellite cell pool, by isolating Pax7-ZsGreen cells from males and females and transplanting into the same or the reciprocal sex. We found no significant differences, i.e., satellite cells from males and females engrafted into the Pax7+ pool to a similar extent. Interestingly we found more cells in the TA of the male recipients compared to female recipients, regardless of the sex of the donor (Figure 2B), a result that correlates with the larger muscle size, and larger endogenous pre-irradiation satellite cell pool size, of the male TA compared to the female [40,41]. We found similar results when we repeated the experiment using NSG-mdx^4Cv^ recipients (Figure 2C), namely that male and female WT Pax7-ZsGreen cells engrafted into the satellite cell pool equally well; however, the average number of Pax7-ZsGreen cells after transplantation of 300 cells into NSG-mdx^4Cv^ recipients was significantly lower than that seen in the WT C57BL6/J recipients, suggesting that the DMD pathology suppresses their ability to contribute, possibly by exposing transplanted cells to a more stressful environment (Figure S1A,B).

### 3.3. Effect of Satellite Cell-Specific Deletion of Esr1 in Males During Homeostasis

**Figure 3:**
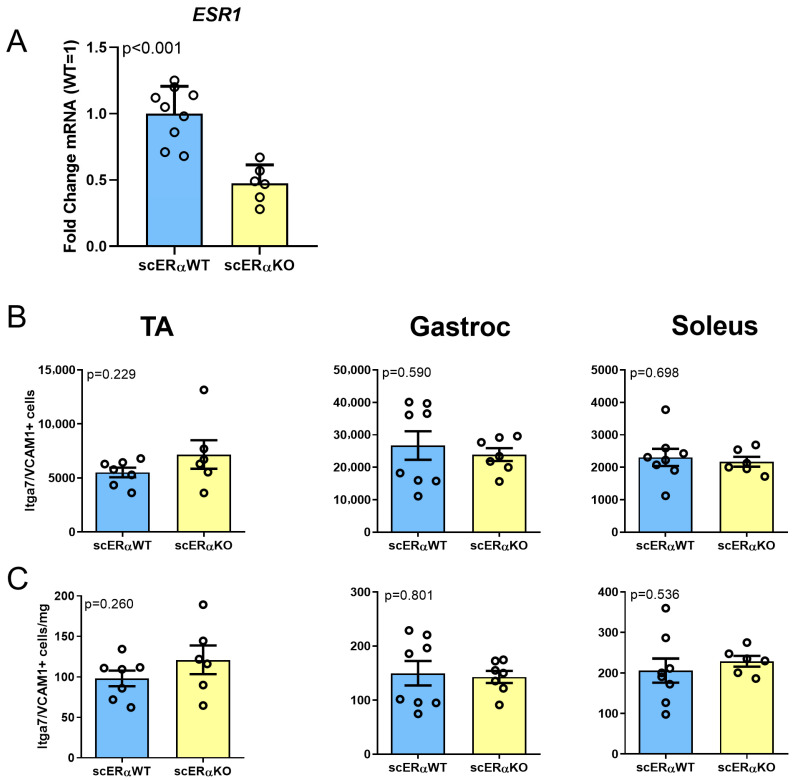
SC-specific deletion of *Esr1* does not deplete the satellite cell compartment in males. (**A**) RTqPCR for Esr1 gene in the VCAM1+/Intga7+ cells from Pax7-cre- or cre+ male mice treated with tamoxifen (*n* = 4). (**B**) Total number of CD45/CD31(Lineage)-negative, VCAM1+/Itga7+-positive cells in tibialis anterior (TA), gastrocnemius (Gastroc), and soleus muscles in Esr1^FL/FL^, Pax7-cre +/− males. (**C**) Density of CD45/CD31(Lineage)-negative, VCAM1+/Itga+ cells in TA, gastrocnemius and soleus in Esr1^FL/FL^, Pax7-cre +/− males. Data are presented as mean ± SEM, *p* values calculated by *t*-test.

To characterize the importance of Esr1 signaling in the satellite cells, we used the inducible satellite cell-specific *Esr1* knockout mouse [32]. Male mice carrying or lacking *Pax7-creERT2* were treated with IP tamoxifen for 5 consecutive days, and two weeks after the last dose of tamoxifen, the hind limb muscles were dissected, enzymatically digested, then stained for satellite cell FACS analysis using the Lin(CD45/CD31)- Itga7+VCAM1+ strategy [47] (Figure S2A). To confirm recombination, we isolated the RNA from sorted cells and tested the expression of the *Esr1*, *GPER*, and *Androgen receptor*, *AR*, which showed a statistically significant decline in the expression of *Esr1* in cre+ animals (*Esr1*-mutant satellite cells) relative to their respective cre-negative controls (Figure 3A). However, when we quantified satellite cells, neither absolute number nor density of satellite cells differed between the cre- and cre+ male mice in the TA, gastrocnemius, and soleus muscles (Figure 3B,C). We tested *Esr1-*knockout females side by side with the males, and found, as expected, a significant decline in the number of Pax7-ZsGreen+ cells in the muscles of cre+ females compared to cre- controls (Figure S2B).

### 3.4. Effect of Satellite Cell Esr1 Ablation on Satellite Cell Pool Response to Injury

**Figure 4:**
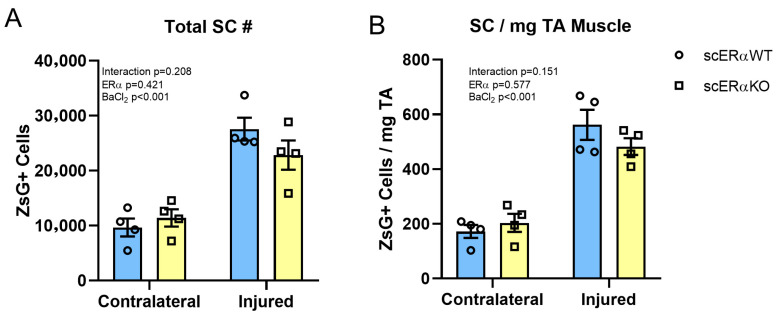
Male scERαKO cells expand normally after muscle injury. (**A**) Total number and Pax7-ZsGreen+ cells in the BaCl_2_ injured and contralateral TA from both Esr1^FL/FL^, Pax7-cre +/− males (*n* = 4); (**B**) Density of Pax7-ZsGreen+ cells in (**A**). Data are presented as mean ± SEM; *p*-values calculated by two-way ANOVA.

To evaluate the necessity of ERα signaling in male satellite cells in the muscle’s response to injury, we unilaterally injured the TA muscles of male mice with BaCl_2_. Twenty-one days following the first injury, a second injury to the same muscle was induced and mice were euthanized 28 days later. The number of Pax7-ZsGreen+ cells was quantified from the injured and contralateral uninjured TA by FACS, revealing a statistically significant expansion of the satellite cells pool after injury, in both the *Esr1* WT and KO mice (Figure 4A,B).

### 3.5. ERα Is Necessary for Male Satellite Cell Self-Renewal in the Female, but Not Male, Environment

**Figure 5:**
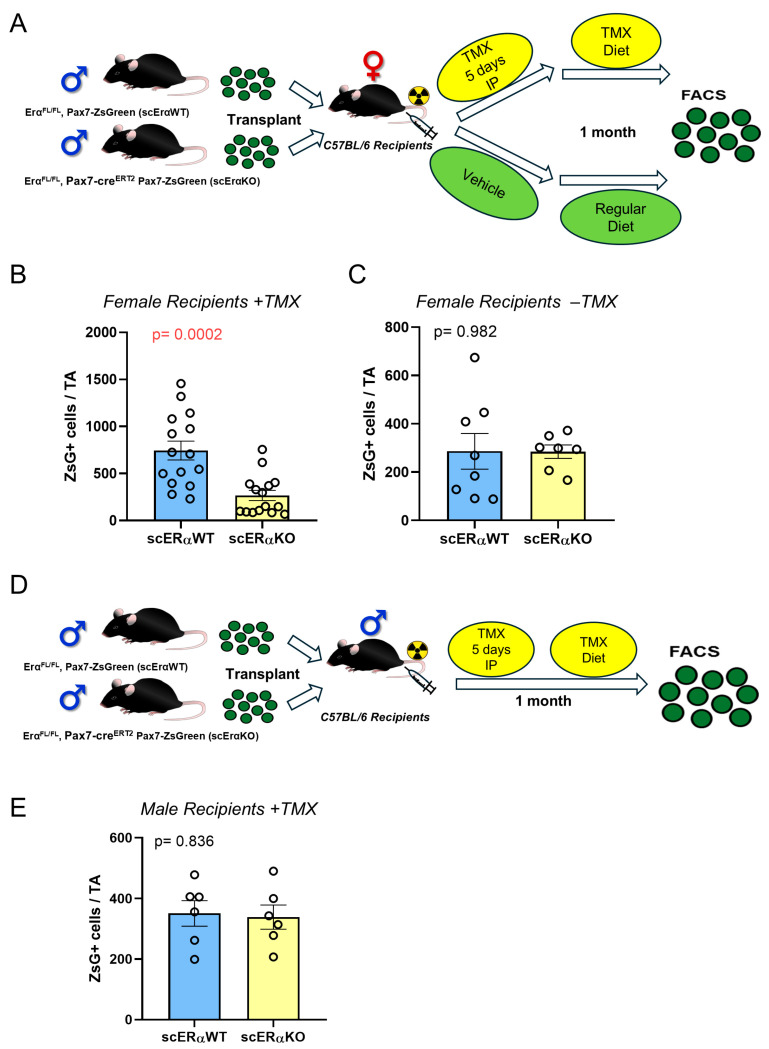
ERα is required in male satellite cells for optimal engraftment into female but not male muscle. (**A**) Schematic for the transplantation experiment into female recipients. (**B**) Total number of Pax7-ZsGreen+ cells in the TA muscle of the female recipients treated with tamoxifen and maintained on tamoxifen diet for 1 month (*n* = 8). (**C**) Total number of Pax7-ZsGreen+ cells in the TA muscle of female recipients treated with sunflower oil vehicle (*n* = 8). Analysis was performed after 1 month. (**D**) Schematic for the transplantation experiment into male recipients. (**E**) Total number of Pax7-ZsGreen+ cells in the TA muscle of the male recipients treated with tamoxifen and maintained on tamoxifen diet for 1 month (*n* = 6). Data are presented as mean ± SEM; *p*-values calculated by *t*-test.

Our previous results found a strong dependence of satellite cell engraftment on the availability of E2 in the host environment and an intact estrogen receptor (ERα, *Esr1*) [32]. We therefore tested whether ERα was necessary in male satellite cells, using the in vivo transplantation assay because it is the most stringent way to evaluate the self-renewal potential of satellite cells, putting tremendous expansion pressure on a small number of donor cells that are also called upon to generate fibers. For these studies, we used 300 Pax7-ZsGreen cells from donors Esr1^FL/FL^; +/− Pax7-CreERT2; Pax7-ZsGreen male mice, such that satellite cells would be marked with green fluorescence if they self-renewed and ERα could be conditionally eliminated only from satellite cells. ZsGreen+ cells were transplanted into irradiated and injured TA muscles of wild-type C57BL/6J females, with the recipients treated with tamoxifen to induce the recombination. The mice were kept on tamoxifen diet throughout the experiment to suppress any deletion escapers (Figure 5A) [18]. We found that deletion of the ERα in male cells significantly reduced their self-renewal potential in the female E2-replete host environment (Figure 5B and Figure S3A). We repeated the same assay with independent donors and the recipient mice were treated with the vehicle only instead, and in this paradigm, we found no difference in the absence of tamoxifen (Figure 5C), supporting that the decline in the self-renewal potential was solely due to ERα deletion.

We then repeated the same transplantation assay using C57BL/6J male recipients instead (Figure 5D), and here we found no difference in the self-renewal between the ERα WT and knockout cells, supporting the interpretation that the dependence of male satellite cells on ERα signaling occurs only in a female environment (Figure 5E and Figure S3B).

## 4. Discussion

Sex-specific differences in adult skeletal muscle including those in satellite cells have been reported [48,49,50,51,52]. While the effects of androgens in maintaining normal muscle physiology in males have been evaluated [53,54], the necessity or activity of estrogens in males is largely obscure.

We have shown in both aged males [41] and females [40] that although satellite cells decline in number in locomotory muscles with age, they do not lose their capacity to engraft and regenerate if transplanted into a young environment. On the other hand, self-renewal of young or old satellite cells is impaired when they are transplanted into an aged female environment, an effect that can be countered to some extent by the provision of hormone (E2) replacement therapy [40]. This demonstrated that non-cell-autonomous environmental factors dominated over cell-autonomous age in driving age-related regenerative deficiencies of satellite cells. We have also shown that in females, E2 is an important factor in setting the satellite cell pool size, with loss of E2 signaling via OVX or satellite cell-specific deletion of *Esr1* leading to 30–50% decline in satellite cell number in most muscles [32]. The current study was undertaken to determine to what extent E2 signaling may be operative in male satellite cells, even though males have much lower levels of systemic E2 than females do. Estrogens are secreted primarily from the granulosa cells of the ovaries in females and the Sertoli cells of the testes in males [30,55], but in both sexes, additional tissues including adipose also endogenously produce estrogens [56,57]. Whether the low levels of E2 in males impact satellite cell maintenance or self-renewal was unknown. Some data exists on the response of male satellite cells to female sex hormones in the context of experimentally elevating E2 levels in males. Studies have reported the beneficial role of exogenously supplied estrogen in preventing disuse muscle atrophy in male rats [58] and a greater extent of 3-day post-injury (downhill running)-induced increase in Pax7+ cells number after estrogen administration, again in male rats [59]. These data do not distinguish between direct responses of satellite cells to estrogen and changes in the environment mediated by systemic estrogen delivery that indirectly impact satellite cells through other pathways; the potential effect of the low endogenous level of E2 in males was also not addressed. They are also not relevant to the hypothesis that male cells respond to locally produced E2 from the action of aromatase in niche cells on circulating testosterone. Here, we show that male satellite cells express *Esr1* at levels equal to or greater than female satellite cells, suggesting the possibility of their direct regulation by E2, either systemic or local.

Our data shows that deletion of *Esr1* in satellite cells has no effect on the homeostatic maintenance of pool size in males, nor does it reduce the expansion in pool size seen after severe BaCl_2_ injury in male TA muscles. The most stringent test of self-renewal potential is arguably the satellite cell transplantation assay, in which 300 satellite cells are delivered into a TA muscle that has been irradiated and injured with BaCl_2_ or cardiotoxin [41,45]. This forces the satellite cells to regenerate a volume of muscle that would normally harbor an order of magnitude more satellite cells than are being transplanted, in addition to repopulating it with the self-renewed pool. Three hundred cells is also within the linear range of this assay, which begins to show severe non-linearity at around 1000 cells or more [41], meaning that at 300 cells it is very sensitive to minor changes in self-renewal potential. Even under this very sensitive assay, we find no difference between *Esr1* KO and WT satellite cells, when transplanted into males. On the other hand, when the same cells were transplanted into females, the *Esr1* KO satellite cells showed a greatly diminished self-renewal potential. This clearly demonstrates that male cells are competent for estrogen signaling, but that it is apparently irrelevant for self-renewal in the normal male environment. These data clearly rule out the hypothesis that male satellite cells respond to local aromatase-generated E2 derived from circulating testosterone. They also rule out a male satellite cell dependence on circulating E2. We propose two possible explanations for the lack of functional effect with loss of *Esr1* in males: (1) the levels of E2, being much lower in normal males, may simply be below the threshold necessary for physiological effect on satellite cells, or (2) another signaling pathway may be operating in males that makes E2 signaling redundant, in which case eliminating that pathway might expose an E2 dependence in males. Previous studies with cross-sex transplants that did not involve hormone signaling manipulation showed that male and female satellite cells produce roughly equivalent numbers of myofibers when transplanted into male or female hosts [43]. If the second proposition is correct, this presumably means that female satellite cells should also be competent to respond to the compensatory signaling pathway operating in males. The possibility that testosterone might constitute such a pathway is supported by the finding that testosterone administration in male subjects leads to elevated PAX7+ cell number [60].

## 5. Conclusions

In summary, these studies demonstrate that estradiol signaling via estrogen receptor alpha is fully functional in male satellite cells, although not necessary in a normal male environment, ruling out the hypothesis that testosterone is converted to E2 by local aromatase to regulate male satellite cells. However, when male satellite cells find themselves in a female environment, they are strongly dependent on E2 for self-renewal, showing that sexually dimorphic mechanisms mediated by the environment regulate satellite cell pool size.

## Data Availability

Data associated with this study will be made available upon reasonable request to Michael Kyba.

## Supplementary Materials

The following supporting information can be downloaded at https://www.mdpi.com/article/10.3390/cells14201606/s1, Figure S1: FACS gating and comparing engraftment in WT vs. NSG-mdx4Cv recipients; Figure S2: FACS gating and normalization by muscle mass of Pax7-ZsGreen counts; Figure S3: Representative FACS plots for transplantation of *Esr1^FL/FL^* male cells.

## Abbreviations

The following abbreviations are used in this manuscript:

AR	Androgen receptor
DMEM	Dulbeco’s modified Eagle medium
E2	17β-Estradiol
EdU	5-ethynyl-2′-deoxyuridine
ER	Estrogen receptor
FBS	Fetal bovine serum
GPER	G-Protein coupled estrogen receptor
KO	Knockout
Lin–	Lineage-negative
MTT	3-(4,5-dimethylthiazol-2-yl)-2,5-diphenyltetrazolium bromide
OVX	Ovariectomy
PBS	Phosphate-buffered saline
PI	Propidium iodide
SC	Satellite cell
scERαKO	Satellite cell *Esr1* knockout
scERαWT	Satellite cell *Esr1* wild-type
SEM	Standard error of the mean
TA	Tibialis anterior (muscle)
WT	Wild-type
