# Supplementary material for: Estrogen Receptor Regulates Male Satellite Cells in a Female, but Not Male, Environment
Source: Cells. 2025 Oct 16;14(20):1606. doi: 10.3390/cells14201606 (PMC12564696; doi:10.3390/cells14201606)
Supplement: Supplementary file 1 [file cells-14-01606-s001.zip › cells-3862159-supplementary.pdf]

Figure S1

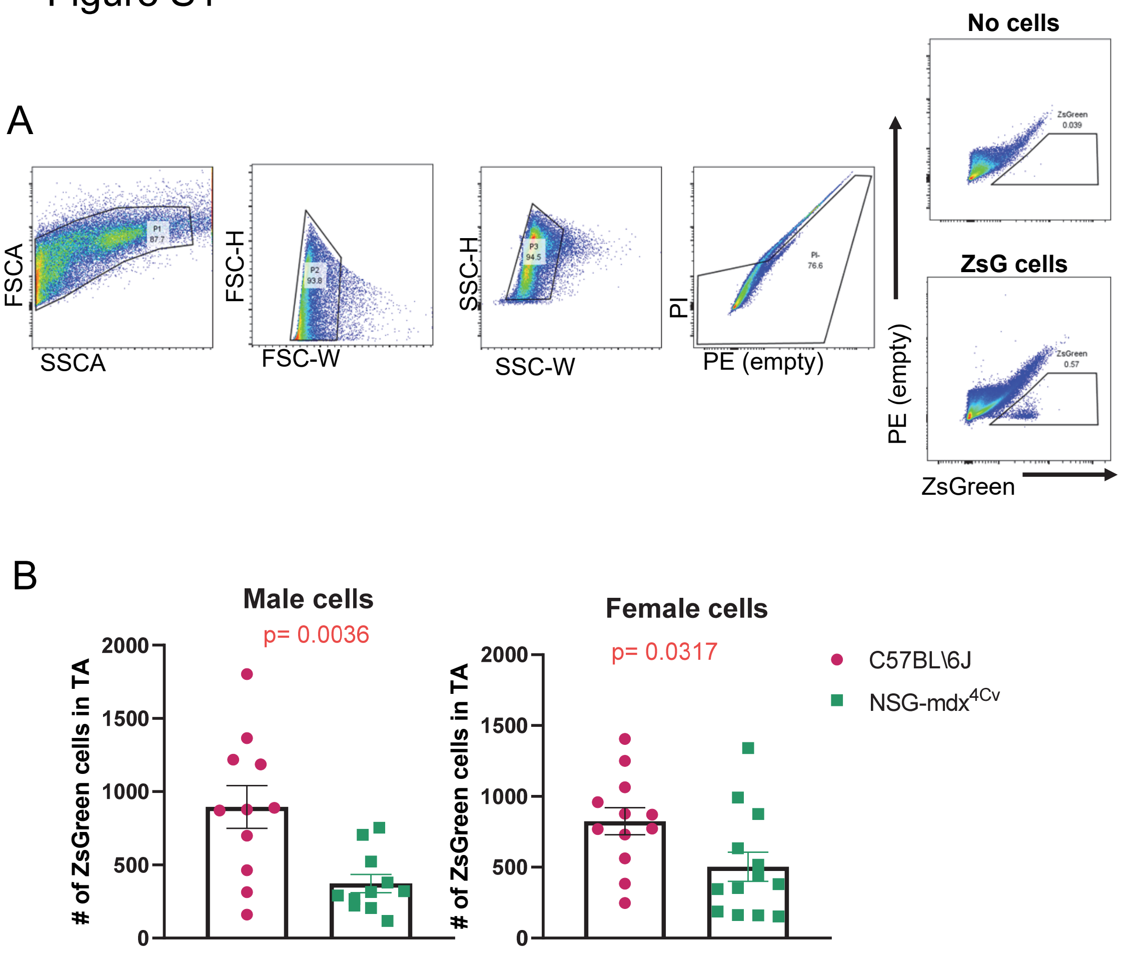

Figure S1. FACS gating and comparing engraftment in WT vs NSG-mdx^{4Cv} recipients

(**A**) Schematic of FACS gating strategy for Pax7-ZsGreen+ cells. PI-negative cells were evaluated on the PE *vs.* green channels.

(**B**) Total number of Pax7-ZsGreen cells in the C57BL/6 and NSG mdx^{4Cv} recipients' TA 1 month after transplanting 300 ZsGreen cells from male and female doners. The data combine all donor cells (male and female) to show average engraftment in WT or NSG-mdx^{4Cv} recipients.

Figure S2

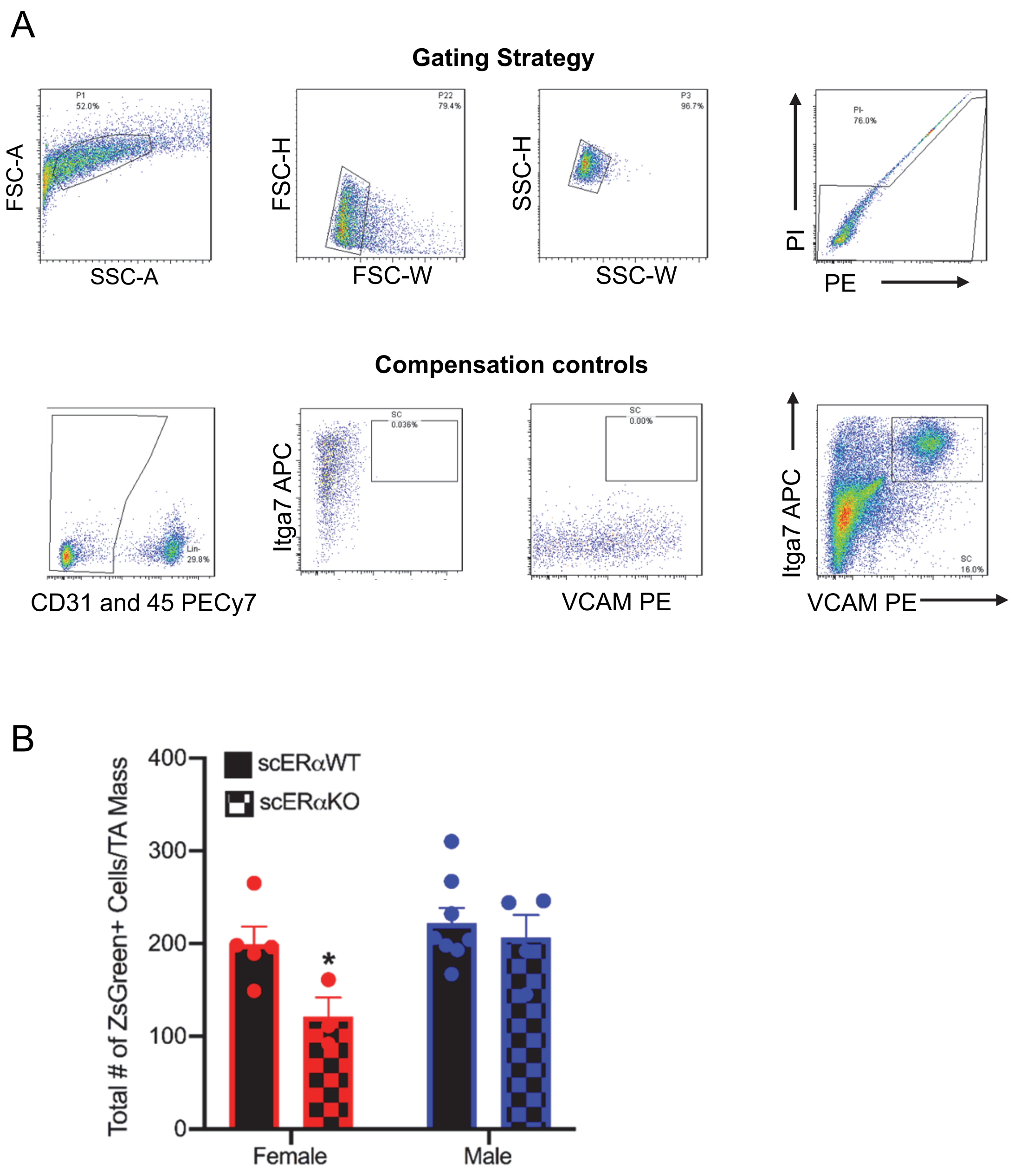

Figure S2. FACS gating and normalization by muscle mass of Pax7-ZsGreen counts

(**A**) Schematic of FACS gating strategy for VCAM1/Itga7+ cells. FACS plots show single stained controls. In the combination panel, the PI-negative cells were evaluated for Itga7 (APC) *vs* CD31/CD45 (PECy7) channels to identify Lin– population. The Lin– cells were subsequently evaluated on the Itga7 (APC) and VCAM1 (PE) to separate the satellite cells

(**B**) Density of Pax7-ZsGreen cells in the TA of *Esr1$^{Fl/Fl}$, Pax7-cre +/-* females and males 2 weeks after Tamoxifen treatment.

Figure S3

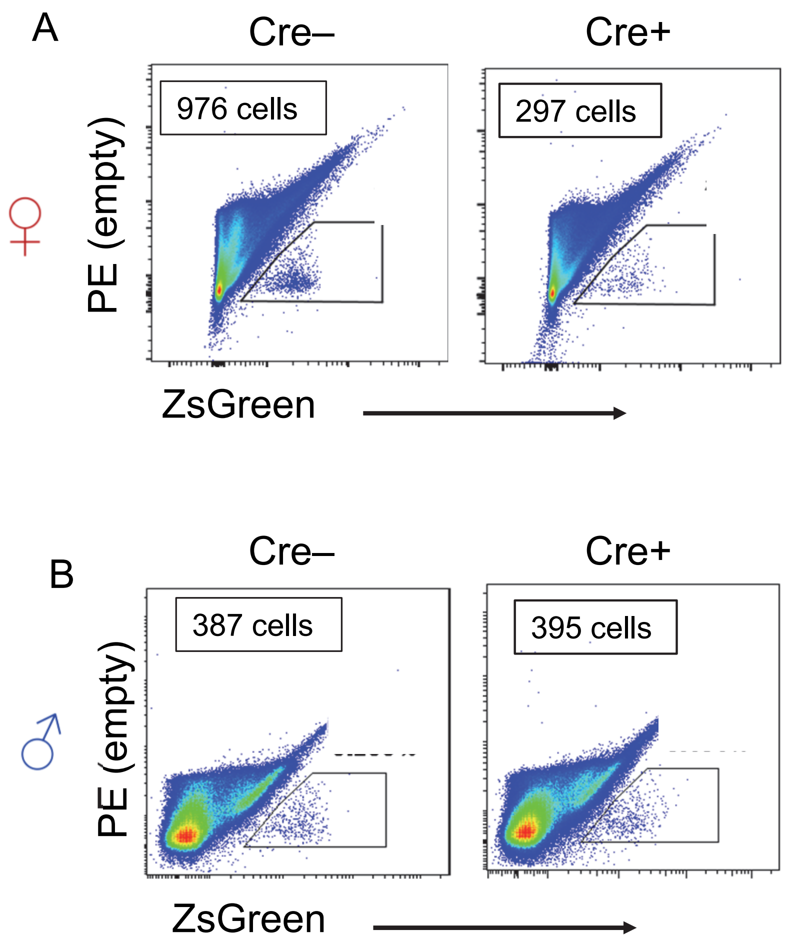

Figure S3. Representative FACS plots for transplantation of *Esr1*$^{FL/FL}$ male cells

(**A**) Representative FACS plots showing number of Pax7-ZsGreen+ cells from *Esr1*$^{Fl/Fl}$*; Pax7-ZsGreen; Pax7-cre +/-* male donors in the TA muscles of female recipients treated with Tamoxifen and maintained on Tamoxifen diet for 1 month.

(**B**) Representative FACS plots showing number of Pax7-ZsGreen+ cells from *Esr1*$^{Fl/Fl}$*; Pax7-ZsGreen; Pax7-cre +/-* male donors in the TA muscles of male recipients treated with Tamoxifen and maintained on Tamoxifen diet for 1 month.
